# Targeting miR-199a reduces fibrosis in hypertrophic cardiomyopathy

**DOI:** 10.1016/j.jmccpl.2023.100057

**Published:** 2023-12-09

**Authors:** Stanislovas S. Jankauskas, Fahimeh Varzideh, Urna Kansakar, Gaetano Santulli

**Affiliations:** a Department of Medicine, Division of Cardiology, Wilf Family Cardiovascular Research Institute, Einstein Institute for Neuroimmunology and Inflammation (INI), Albert Einstein College of Medicine, Montefiore University Hospital, New York, NY 10461, USA; b Department of Molecular Pharmacology, Einstein-Mount Sinai Diabetes Research Center (ES-DRC), Fleischer Institute for Diabetes and Metabolism (FIDAM), Einstein Institute for Aging Research, Albert Einstein College of Medicine, Montefiore University Hospital, New York, NY 10461, USA

Hypertrophic cardiomyopathy (HCM) is a complex cardiovascular disorder characterized by abnormal thickening of the heart muscle. It is a genetic condition with autosomal dominant inheritance. HCM is a leading cause of sudden cardiac death in young individuals and athletes.

HCM is clinically under-recognized and according to recent data [1], the estimate of its prevalence in the general population based on echocardiography and cardiac magnetic resonance imaging (CMR) is ~1 in 500, indicating that ~15 million people worldwide could be affected by this disease, including as many as 700,000 Americans.

HCM is characterized by asymmetric hypertrophy of the left ventricle, where the muscle wall becomes abnormally thickened. This hypertrophy is a result of genetic mutations affecting proteins in the cardiac sarcomere, the contractile unit of the heart muscle [2]. The majority of these mutations are inherited in an autosomal dominant fashion, meaning that an individual only needs one copy of the mutated gene from either parent to develop the condition. Not all individuals with HCM have a known family history of the condition, as *de novo* mutations or incomplete penetrance can occur [3]. The most commonly affected genes in HCM are those that encode proteins involved in the sarcomere structure [4]. Mutations in these genes disrupt the normal function of the sarcomere, eventually leading to abnormal thickening of the heart muscle that can cause dynamic left ventricular outflow tract obstruction, impairing blood flow from the heart.

## 1 Fibrosis in HCM

Fibrosis plays a significant role in the pathophysiology of hypertrophic cardiomyopathy (HCM). The presence of fibrosis in the heart can have several implications for the structure and function of the myocardium in individuals with HCM. Indeed, fibrosis disrupts the normal architecture of the myocardium, impairing the coordinated contraction and relaxation of the muscle. Fibrosis also contributes to increased myocardial stiffness: as collagen accumulates in the myocardium, it reduces the compliance of the heart muscle, making it less elastic; such increased stiffness can affect the heart's ability to fill with blood during diastole, leading to impaired relaxation and potentially contributing to diastolic dysfunction [5]. These aspects can lead to inefficient pumping of blood and contribute to the symptoms associated with HCM, such as dyspnea and fatigue. Importantly, fibrosis creates a substrate for the development of arrhythmias. The abnormal tissue structure, combined with alterations in electrical conductivity, can create areas of slow conduction and promote the formation of reentrant circuits, increasing the risk of atrial and ventricular arrhythmias and sudden cardiac death [6].

## 2 MicroRNA-199a

MicroRNA-199a (miR-199a) is a small non-coding RNA molecule that plays a regulatory role in gene expression. MicroRNAs are short RNA sequences, typically consisting of around 20–22 nucleotides, and they are involved in post-transcriptional gene regulation: they exert their regulatory function by binding to messenger RNA (mRNA), leading to its degradation or to the inhibition of its translation into protein. The specific genes targeted by miR-199a can vary, and the regulatory roles of miR-199a are highly context-dependent. In the context of cardiovascular biology, miR-199a has been implicated in various processes, including cardiac development, angiogenesis, and responses to cardiac stress and injury. For instance, it is involved in the regulation of cardiac hypertrophy: dysregulation of miR-199a expression has been observed in hypertrophic hearts, and may contribute to the pathological remodeling associated with cardiac hypertrophy [7].

The expression of miR-199a has been found to be altered in response to ischemic heart disease. It may participate in the molecular responses to ischemic injury and influence the repair and remodeling processes in the heart following damage. Intriguingly, in seminal studies, Serena Zacchigna and Mauro Giacca had elegantly demonstrated that miR-199a-3p is able (alongside miR-590-3p) to stimulate proliferation of cardiomyocytes and, once expressed in the mouse heart using viral vectors or synthetic miRNA-lipid formulations, induce cardiac regeneration after myocardial infarction [8,9]. However, when performing the experiments in pigs, the persistent and uncontrolled expression of miR-199a-3p resulted in sudden arrhythmic death of most of the treated animals [10]; these events were concurrent with myocardial infiltration of proliferating cells displaying a poorly differentiated myoblastic phenotype [10].

## 3 Inhibiting miR-199a attenuates fibrosis in HCM

**Fig. 1:**
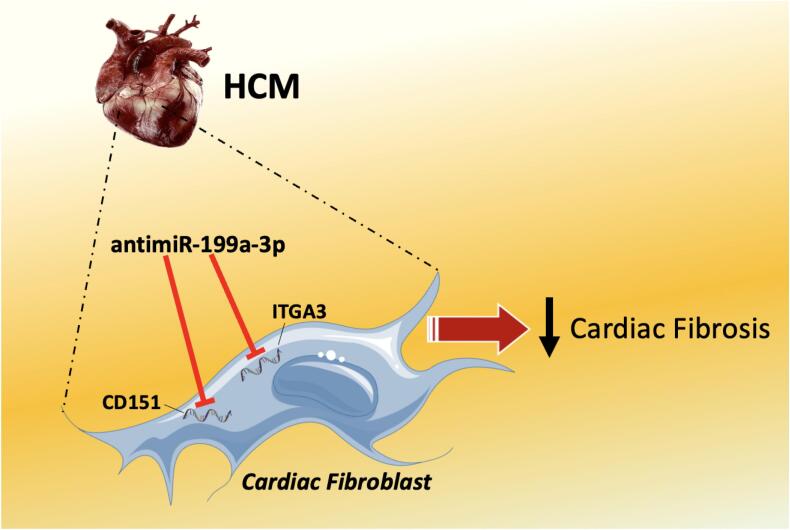
The inhibition of miR-199a-3p decreases fibrosis in hypertrophic cardiomyopathy. HCM: hypertrophic cardiomyopathy; ITGA3: Integrin Subunit Alpha 3.

Testing different mouse models of HCM, in this issue of *JMMC Plus*, Zalivina and collaborators demonstrate that miR-199-3p is consistently upregulated in several murine models of HCM [11]. Then, they transfected murine cardiac fibroblasts with miR-199a-3p or control mimics and, through an unbiased proteomics approach of the conditioned media, they observed that numerous extracellular matrix (ECM) proteins were differentially secreted. To examine the actual role of miR-199a-3p *in vivo*, the Authors inhibited its function using lock-nucleic acid (LNA)-based inhibitors in an established mouse model of HCM, showing that the progression of cardiac fibrosis was markedly mitigated when inhibiting miR-199a-3p. CD151 and ITGA3, known to regulate ECM remodeling [12,13], were identified as targets of miR-199a-3p (Fig. 1).

Overall, these findings are consistent with previous observations showing that the injection of adeno-associated virus (AAV)-mediated anti-miR-199a tough decoys significantly alleviated cardiac hypertrophy by targeting PGC1α [14], a master regulator of mitochondrial biogenesis. Similarly, other studies had demonstrated that miR-199a impairs autophagy and induces cardiac hypertrophy through mTOR activation [15,16].

The main limitation of the study, in addition to the ones acknowledged by the authors, is the lack of experimental evidence in human settings (*e.g.* human cardiac cells). It is important to note that the understanding of the exact functions of miR-199a in the cardiovascular system is continually evolving, and ongoing research is exploring its precise roles and regulatory mechanisms. Additionally, the dysregulation of miR-199a has been implicated in various pathological conditions beyond the cardiovascular system, including cancer and metabolic disorders [[17], [18], [19], [20], [21]].

Understanding the contribution of fibrosis in HCM is critical for comprehensively managing this complex condition. Research is ongoing to explore therapeutic strategies targeting fibrosis, with the aim of improving outcomes for individuals with HCM. Early detection of fibrosis through advanced imaging techniques may also have implications for risk stratification and treatment planning in HCM patients.

## Declaration of competing interest

None.
